# A hybrid broadband metalens operating at ultraviolet frequencies

**DOI:** 10.1038/s41598-021-81956-4

**Published:** 2021-01-27

**Authors:** Farhan Ali, Serap Aksu

**Affiliations:** Department of Physics, Koc University, 34450 Istanbul, Turkey

**Keywords:** Optics and photonics, Physics

## Abstract

The investigation on metalenses have been rapidly developing, aiming to bring compact optical devices with superior properties to the market. Realizing miniature optics at the UV frequency range in particular has been challenging as the available transparent materials have limited range of dielectric constants. In this work we introduce a low absorption loss and low refractive index dielectric material magnesium oxide, MgO, as an ideal candidate for metalenses operating at UV frequencies. We theoretically investigate metalens designs capable of efficient focusing over a broad UV frequency range (200–400 nm). The presented metalenses are composed of sub-wavelength MgO nanoblocks, and characterized according to the geometric Pancharatnam–Berry phase method using FDTD method. The presented broadband metalenses can focus the incident UV light on tight focal spots (182 nm) with high numerical aperture ($\hbox {NA}\approx 0.8$). The polarization conversion efficiency of the metalens unit cell and focusing efficiency of the total metalens are calculated to be as high as 94%, the best value reported in UV range so far. In addition, the metalens unit cell can be hybridized to enable lensing at multiple polarization states. The presented highly efficient MgO metalenses can play a vital role in the development of UV nanophotonic systems and could pave the way towards the world of miniaturization.

## Introduction

The demand for compact optical devices in daily life has initiated the need to miniaturize the conventional optical components. As any miniaturization, realizing smaller optical components with identical optical properties has been challenging, and needs a thorough research on materials, design and fabrication. Using uniformly spaced sub-wavelength nanoblocks, metasurfaces can enable a scale down on device sizes, and enable even superior quality optical responses by minute control of the amplitude, phase, polarization and dispersion of light^1–5^. Recently metasurfaces have been utilized for a large variety of applications such as metalenses^6–12^, meta-holograms^13–18^, polarizers^2,19,20^. Among these, metalenses received particular attention, aiming to replace the traditional bulky and expensive objective lenses with ultra-thin, flat, light-weighted, and cost effective nanostructures without losing lens’s performance at any relevant frequency^21^.

Realization of metalenses in optical wavelengths has been challenging and depends highly on the refractive index of the materials. As the metals are mostly preferred for metasurfaces, the preliminary metalens designs utilize metallic nanoblocks or holes^22,23^. They paved the way to understand phase engineering between the nanostructures in the infrared region, however, due to the intrinsic absorption losses, metallic metalenses could be not be extended for operations in shorter wavelengths. Instead, dielectric materials with relatively higher refractive indices (n>2) and relatively negligible absorption loss has been used for metalenses to enable high focusing efficiency in ultraviolet (UV)^11,24,25^ and visible (VIS)^7,26–28^ frequencies. So far the studies rely on the fact that efficient metalenses require sharp refractive index contrast against the background for the efficient handling of phase. There has been preliminary attempts to understand the role of the refractive index on metalens performance^29,30^, but still there is no robust concrete calculation about the refractive index requirements for the efficient lenses. On the other hand there is a relation between the focusing efficiency of a metalens and the transparency of the materials used. A transparent material has to be used for efficient focusing of the incident light. Large band gap materials provide the required optical transparency and Moss relation ($\hbox {n}^4$ $\hbox {E}_g=95\,\hbox {eV}$), and Ravindra relation (n = 4.16–0.86 E$_g$)^31^ states that the band gap and refractive index is inversely related. Thus, low refractive index materials are needed to realize metalenses with high focusing efficiency. This creates a problem particularly in UV frequencies where available transparent materials have limited range of dielectric constants. Previous studies on UV metalenses mostly utilize AlN or Si$_3$N$_4$^11,25^. These materials are transparent in UV, respectively having 6 and 5 eV band gaps, and 2.28 and 2.17 refractive indices at 300 nm wavelength. The optical constants of these materials fits the requirement of efficient UV metalenses, however the presented focusing efficiencies so far have been limited to ~ 77% at most^11,24,25^. Moreover, the previous studies typically use the nanoblocks with varying sizes to enable multi-wavelength focusing behavior. In addition, the focusing capacity under broadband illumination or polarization independence at broad UV range has not been studied in detail.

In this letter, we report a highly efficient theoretical metalens design composed of magnesium oxide (MgO) nanoblocks operating at the wide UV spectral range of 200–400 nm without the need for changing the nanoblock dimension. MgO enables both the polarization conversion efficiency (PCE) and focusing efficiency (FE) of the designated metalenses to reach ~ 94%, which is the best value reported in this spectral range. The proposed metasurfaces are characterized with Pancharatnam–Berry (P–B) phase method by rotation of the nanoblocks to achieve full 2$\pi$ phase coverage for circular polarized light sources. In addition unlike any previous study in the UV range, a hybrid unit cell structure is formed by meeting two identical nanoblocks in a unit cell with a certain configuration satisfying the best PCE. The proposed hybrid metalens enables focusing of both left handed circular polarized (LCP) and right handed circular polarized (RCP) incident light with the same FE. Furthermore, the lenses have high numerical aperture (NA) (~ 0.8), and are capable of focusing light to very tight focal spots (182 nm). The demonstrated metalens enables new flat optics in UV, and MgO is an ideal material for metalenses operating at shorter optical wavelengths.

**Figure 1:**
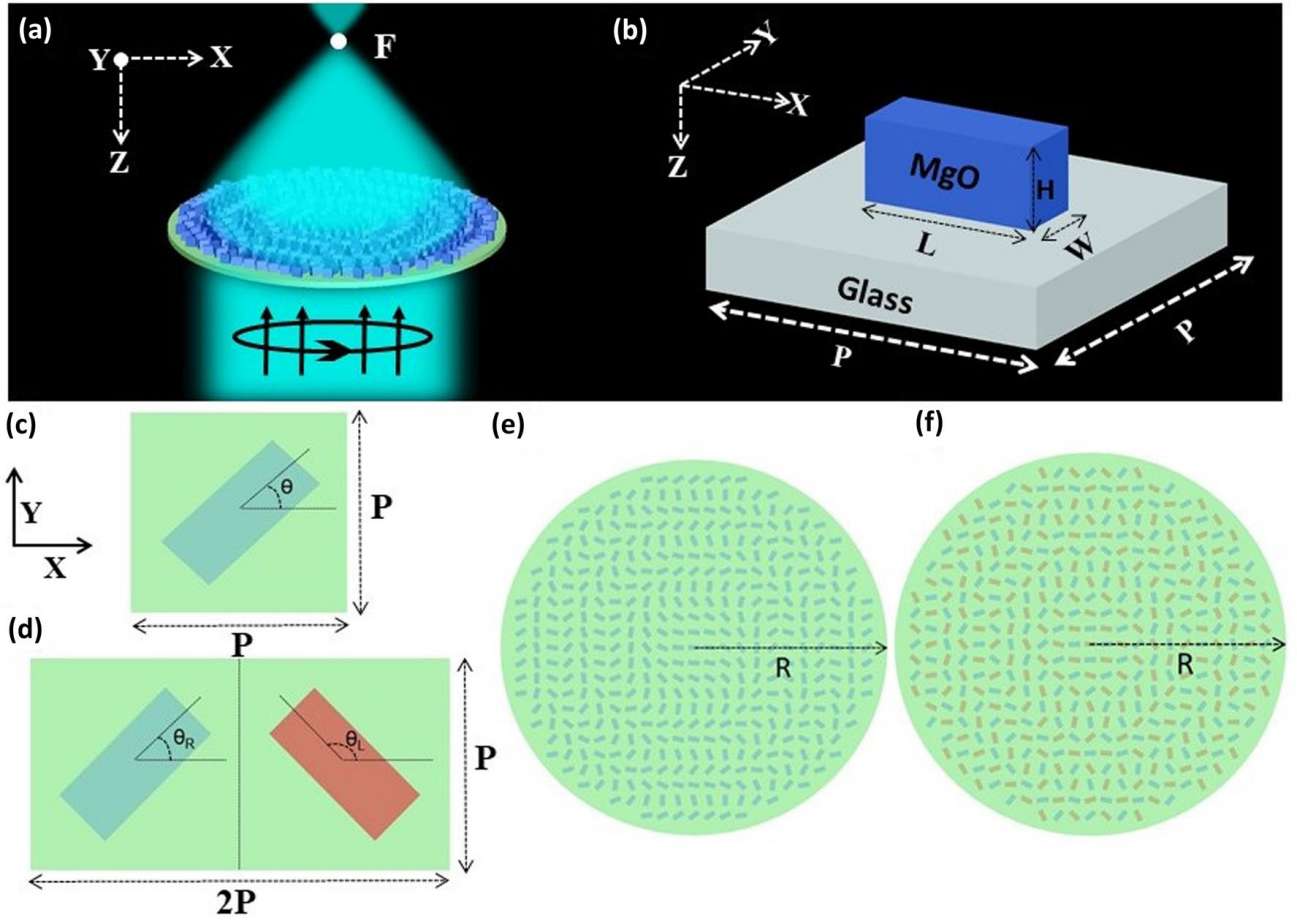
A schematic view of the designed metalens focusing circular polarized incident light at focal point *F* (**a**), and its MgO building block on glass substrate with height *H*, length *L*, width *W*, and unit cell has the dimensions $P\times P$ (**b**). (**c**) The unit cell of a simple metalens, rotated by an angle $\theta$ to impart required phase according to geometric P–B phase method. (**d**) Unit cell of a hybrid metalens, where two different colored nanoblocks are rotated by an angle $\theta _R$, $\theta _L$ simultaneously to focus RCP and LCP incident lights. (**e**,**f**) Top view of the simple, and hybrid metalens of radius R.

## Working principle and design of the metalens

To realize a highly efficient and broadband metalens in the UV regime, magnesium oxide (MgO) nanoblocks on a glass substrate are considered as shown in Fig. 1. We adopted MgO due to its high transmission, ultra-wide bandgap (~ 7.8 eV), and suitable refractive index (1.96–1.76) in the designed frequency region (200–400 nm)^32,33^. To focus a circular polarized incident beam, metalens is constructed in such a way that it fulfils the P–B phase condition^34^

1 $$\begin{aligned} \phi _{PB}(x,y) = \frac{2\pi }{\lambda _d}\left( f-\sqrt{f^2+x^2+y^2}\right) \end{aligned}$$

where $\lambda _d$ is the designed wavelength, *f* is the designated focal length set as 7 $\upmu$m and (*x*, *y*) are the corresponding coordinates of each nanoblock. This P–B phase (also called the geometric phase) can be achieved by rotating each nano-element with an angle

2 $$\begin{aligned} \theta (x,y) =\pm \frac{1}{2}\phi _{PB}(x,y)= \pm \frac{\pi }{\lambda _d}\left( f-\sqrt{f^2+x^2+y^2}\right) \end{aligned}$$

for RCP/LCP (+/−) incident light accompanied by reversing the polarization state of the incident beam. In order to obtain high PCE, which is defined as the power ratio of transmitted circular light with opposite helicity to the incident light, each element should act as a half wave plate^6^. This half wave plate feature actually comes from the birefringence property due to the asymmetric cross-section of the nanoblock^35^. Thus, by properly optimizing the geometrical parameters (length *L*, width *W*, height *H*) and period *P* of the unit cell (Fig. 1b), we can achieve maximum PCE resulting in a highly efficient metalens. Optimized structural parameters of the metasurface’s unit cell are $L=180\,\text {nm}, W=75\,\text {nm}, H=900\,\text {nm}$ and $P=200\,\text {nm}$. Due to the rotation the distance between each nanoblock becomes greater than $20\,\text {nm}$ which eases the fabrication of structures with conventional methods. Keeping the nanoblocks identical, two distinct unit cells are utilized in this work, a simple and a hybrid one (Fig. 1c–f). The simple unit cell is composed of a single nanoblock which operates at single polarization, whereas the hybrid unit cell includes two identical nanoblocks that are rotated by an appropriate angle, and it operates at multiple polarizations.

**Figure 2:**
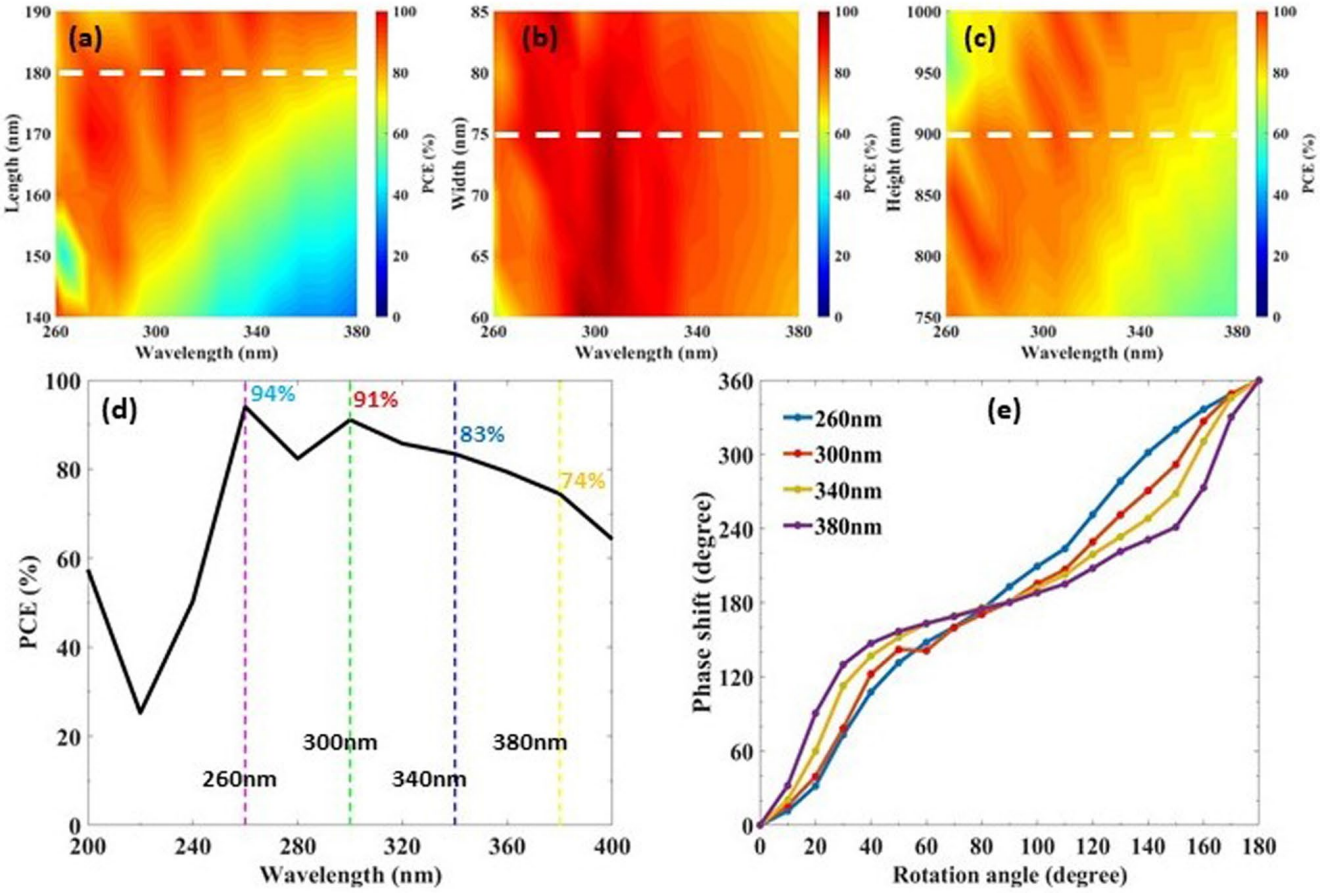
Polarization conversion efficiency (PCE) as a function of incident light wavelength and length (**a**), width (**b**), height (**c**) of the nanoblock. Horizontal dashed lines in (**a**–**c**) represent the optimized parameters (*L*, *W*, *H*) resulting in maximum PCE. (**d**) The simulated PCE of MgO nanoblock on a glass substrate as a function of wavelength. Vertical dashed lines represent the designated four different wavelengths (260 nm, 300 nm, 340 nm, 380 nm) that are chosen to characterize broadband metalens properties, with a minimum PCE of 74%. The optimized structural parameters of nanoblocks are $L=180$ nm, $W=75$ nm, $H=900$ nm, and unit cell with period $\hbox {P}=200$ nm. (**e**) Calculated phase profile of nanoblocks as a function of rotation angle $\theta$ within range of 0$^\circ$–180$^\circ$, at the designated wavelengths.

A commercial electromagnetic solver software based on finite-difference time-domain method (Lumerical Inc., Vancouver, Canada) is employed to perform all simulations. The optical constants of glass and MgO are taken from Palik, Stephens et al. respectively^33,36^. We calculate the refractive index of MgO down to 200 nm using a dispersion relation^37^. After calculating the refractive index within 200–400 nm, we import the data to Lumerical (Figure S1). For the calculations related to the unit cell the incident light source is set as RCP or LCP, periodic boundary conditions are imposed on the *x* and *y* directions, and perfectly matched layer (PML) boundary condition is imposed on the *z* direction. Initially a unit cell is simulated to obtain the PCE of the proposed structure. In addition to the unit cell period (*P*), we optimized the length (*L*), width (*W*), and height (*H*) of the MgO nanoblocks sitting on a glass substrate to achieve maximum PCE as shown in Fig. 2a–c. Meanwhile, in the optimization process of the nanoblock’s length, width, and height, all other geometric parameters are kept constant at their optimized values. Figure 2d shows the calculated PCE across the UV spectrum, which reaches maximum value of $94\%$ at $260\,\text {nm}$. To characterize the broadband properties of the proposed metalens unit cell, we illuminate the metalens with light covering all 200–400 nm frequency range. Within this range four wavelengths are specified to compare the metalens behaviour: $\lambda _d = 260, 300, 340, 380\,\text {nm}$ each of which have different PCE’s. These four different metalens patterns are achieved using Equation 2 and utilize the identical optimized nanoblocks. The phase shift that is observed after the rotation of the nanoblock within an angle of $0-\pi$ is shown for the designated wavelengths in Fig. 2e. The figure shows that at all wavelengths the rotation of the nanoblock by an angle $\theta$ = 0 to 180 degree covers the whole 360 degree phase shift by following the P–B phase condition $\phi _{PB} =2\theta$. Since the same nanoblock design is used for a variety of wavelengths, nanoblocks do not act as a perfect half-wave plate at each wavelength which results in small deviations from the linear phase-angle relationship ($\phi =2\theta$) in Fig. 2e. The broadband metalens using the same nanoblock design does not significantly affect the metalens focusing characteristics at different wavelengths as full $2\pi$ phase coverage is still justified.

After calculating PCE and phase shift on the unit cell, we study the unit cell array. For the unit cell array case, the simulations are performed using the FDTD method by running a script code. The script enables the placement of nanoblocks at each position (x,y) within the designed diameter of the metalens by rotating them according to the P–B phase condition (equation 1). PML boundary condition is employed in all three *x*, *y*, and *z* directions. As stated before in this work we utilized two unit cells, a simple and hybrid metalens. Simple metalens is composed of equally spaced (with period *P*) MgO nanoblocks with the same geometrical parameters (*L*, *W*, *H*). These nanoblocks are rotated by an angle $\theta$ (equation 2) at position (*x*, *y*) to focus incident circular polarized light beam at the focal point of the metalens. The rotation angle of the building block at any point (*x*, *y*) can be determined by the P–B phase (equation 2), which could be an arbitrary value $0-\pi$. After transmitting through the metalens the incident light will be exposed to a phase shift equal to double the rotation angle $\phi =2\theta$, hence achieving the whole 0-2$\pi$ phase coverage. However, this type of metalens depends only on the rotation angle $\theta (x,y) =\sigma \phi (x,y)/2$ of the constituent elements. Hence it works only for either LCP ($\sigma = -1$) or RCP ($\sigma = +1$) incident light. To overcome the polarization limits, following the work of H. Dong et al.^38^, we designed a hybrid metalens for the first time in the UV spectral region. Here, nanoblocks are simultaneously rotated by $\theta _R = +\phi (x,y)/2$ (for RCP) and $\theta _L = -\phi (x,y)/2$ (for LCP) via alternative distribution of the unit cells in both *x* and *y* directions. Hence, by adopting such a technique, the designed metalens can effectively operate to focus both LCP and RCP light.

**Figure 3:**
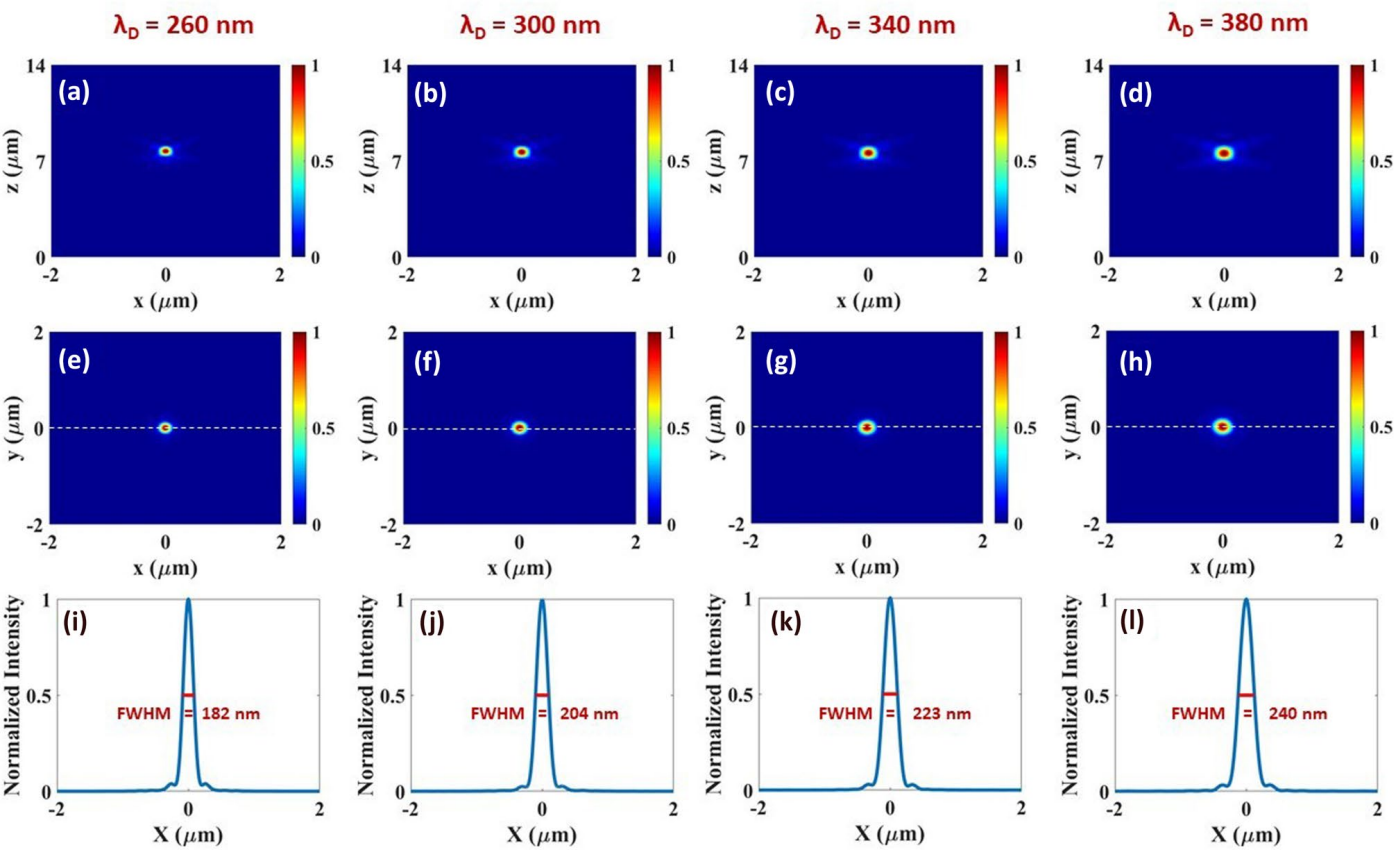
(**a**–**d**) Simulated normalized intensity profiles of focal spot at $y=0$ ($x-z$ plane) for simple metalens at the designated wavelengths $\lambda _d=260, 300, 340, 380\,\text {nm}$ respectively. (**e**–**h**) Simulated normalized focal spot intensity profiles at $z=f$ ($x-y$ plane) for the corresponding wavelength. (**i**–**l**) Normalized intensity profiles of focal spots presented at (**e**–**f**) along the dashed horizontal cut (at $y=0$) for the corresponding wavelength. The profiles present full width at half maximum of $182, 204, 223, 240\,\text {nm}$ respectively. Each metalens is illuminated by monochrome RCP incident light. All the metalenses have $20\,\upmu$m diameter, and the numerical aperture is calculated as (NA$\approx 0.8$) at each wavelength.

## Results and discussion

For the simple metalens, we verify the broadband behavior of the system at the designated four wavelengths $\lambda _d=260\,\text {nm}, 300\,\text {nm},$ $340\,\text {nm}, 380\,\text {nm}$. All the metalenses have a diameter of $20\,\upmu$m and are illuminated by an RCP incident light from the glass side of the substrate as shown in Fig. 1a. Figure 3 shows the focusing characteristics of the simple metalens at different wavelengths in the UV range. The normalized intensity distributions in the $x-z$ plane at $y=0$ are depicted in Fig. 3a–d, whereas intensity profiles at focal point in the $x-y$ plane are shown in Fig. 3e–h. The calculated intensity at each point is normalized with dividing by the maximum field intensity. All the focal spots are highly symmetric and strongly confined at the center of the focal region. Figure 3i–l shows the relevant horizontal cut ($x-y$ plane at $\hbox {y}=0$) over the focal spot. The diffraction limited (~ $\frac{\lambda _d}{2 \text {NA}}$) focal spot exhibit full width at half maximum (FWHM) of $182, 204, 223, 240\,\text {nm}$ respectively. Focal lengths for the four designed metalens are measured as $f=7.75, 7.64, 7.59$, and $7.56\,\upmu$m respectively with a similar $NA\approx 0.8$. Numerical aperture is calculated using the relation $\text {NA} = \sin \left[ \tan ^{-1} \left( R/f\right) \right]$^39^. Similar focusing characteristics with a small deviation ($<3\%$) in the focal lengths strengthen the design’s capacity to operate at multiple wavelengths within broadband UV range (200–400 nm). The depth of focus (DOF) values are calculated using the formula $\Delta f= 4 \lambda f^2/D^2$ and found as 156, 175, 196, 217 nm respectively^40^.

**Figure 4:**
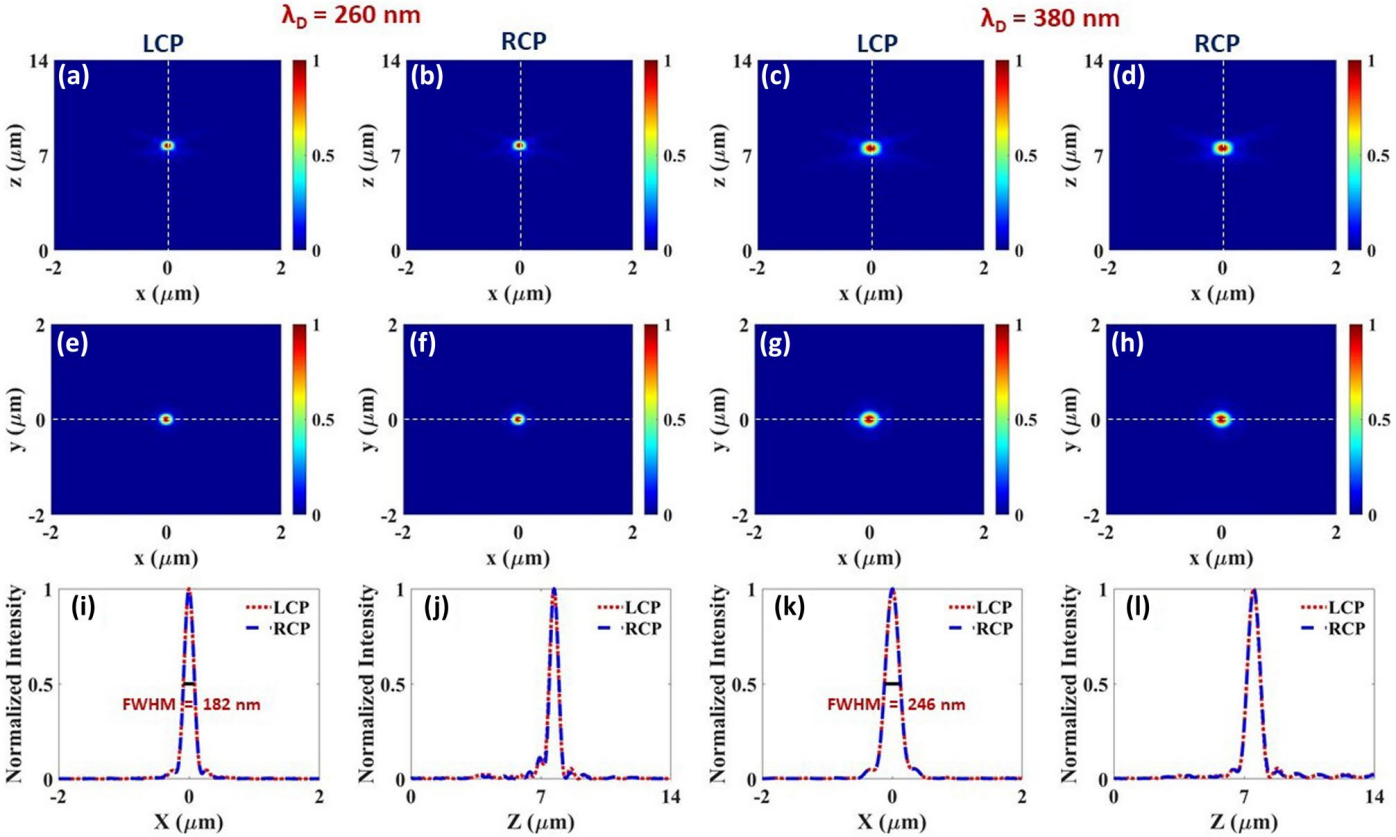
(**a**–**d**) Normalized intensity profiles of the focal spot of the hybrid metalens at $x-z$ plane ($y=0$) for $\lambda _d=260$ nm and 380 nm under both LCP/RCP illuminations respectively. (**e**–**h**) Normalized intensity profiles of the focal spot of the hybrid metalens at $x-y$ plane ($z=f$) at $\lambda _d=260$ nm and 380 nm under LCP, RCP illumination respectively. (**i**,**k**) depicts the intensity profile at the corresponding horizontal dashed lines shown at (**e**–**h**), at $y=0$, giving similar FWHM values under both LCP and RCP polarization. (**j**,**l**) shows the intensity profile at the corresponding vertical dashed lines shown at (**a**–**d**), at $x=0$. All the designed metalenses have the same diameter of $20\,\upmu$m and NA$\approx 0.8$.

Furthermore, the hybrid metalens is investigated at the designated wavelengths of $\lambda _d=260$ nm and 380 nm. The geometric parameters of the unit cell and the number of nanoblocks are kept same as the simple metalens, and the diameter of the metalens is 20 $\upmu$m. The structures are illuminated by both RCP and LCP light. Figure 4a–h show the normalized intensity profiles of the focal spot in the ($x-z$ plane), and ($x-y$ plane) respectively for the hybrid metalens. As in the case of the simple metalens discussed earlier, focal spots are highly focused at the central positions for both polarization states. Figure 4i,k shows that the simple and hybrid metalenses have similar normalized intensity profiles at $z=f$ with similar $\hbox {FWHM}=182$, 246 nm at the corresponding frequencies. Moreover, the measured focal lengths are similar for the simple and hybrid metalenses ($f=7.75, 7.56\,\upmu$m) shown by the normalized intensity profiles as a function of z in Fig. 4j,l. Figure 4j,l displays an insignificant deviation of symmetry for the intensity profiles of the focal spot on x-z plane. This asymmetry might arise a concern of spherical aberration, but it is proven that for normal incident light the flat lenses are free from spherical aberrations and distortion^41^. Our studies and the previous articles show that this asymmetric behavior is in parallel with the mismatch between the designated and measured focal lengths (7 vs. 7.56 $\upmu$m). As the mismatch between the focal lengths grow, the intensity profile on x-z becomes more asymmetric and focusing spreads dramatically^10,38^. Hence, by utilizing the hybrid metalens structure we can achieve highly focused, intense focal spots with diffraction limited FWHM’s under both polarization states without loosing any optical quality for each polarization.

**Figure 5:**
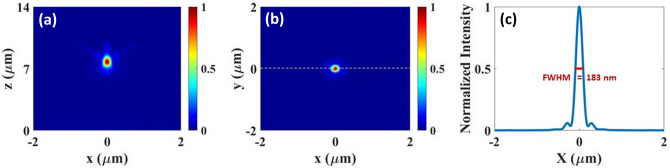
Focusing characteristics of the simple metalens when illuminated by an RCP broadband incident light beam ($\lambda =200$–400 nm). Normalized intensity profiles of focal spot (**a**) ($x-z$ plane) at $y=0$, (**b**) ($x-y$ plane) at $z=7.76\,\upmu$m, (**c**) corresponding horizontal cut at $y=0$ shows FWHM of 183 nm.

Moreover, in order to investigate the effect of broadband and monochromatic illumination source on calculations, we test the previously discussed simple metalens using a broadband illumination source, unlike any previous UV range metalens studies^11,24,25,42^. Keeping all the geometric parameters same, we illuminate the $\lambda _d=300$ nm simple metalens having 10 $\upmu$m diameter with a broadband RCP source ($\lambda =200$–400 nm) and a monochromatic light source at 300 nm. Simulations under the broadband illumination needs more powerful computational resources. Hence, the diameter of the $\lambda _d=300$ nm simple metalens is reduced to 10 $\upmu$m unlike previously discussed one. Every other structural parameters are kept same. Figure 5a,b shows the normalized intensity distribution in the $x-z$ plane at $y=0$, and in the $x-y$ plane at $z=f$ . From the horizontal cut of the intensity profile at the focal spot, FWHM of the simple metalens illuminated with broadband source is found as 183 nm (Fig. 5c). When the same metalens illuminated with a 300 nm monochromatic source, we obtain FWHM of 208 nm. This is an interesting result, showing that under broadband illumination we obtain a tight focusing, similar to a monochromatic illumination. In addition, the focal length of the same metalens under broadband illumination is measured as $\hbox {f}=7.76\,\upmu$m, where as measured as $f=4.64\,\upmu$m for monochromatic one. This result and additional investigations show that illuminating the metalens with the broadband light source changes the focal length (see Supplementary Information) compared to the monochromatic illumination. To analyze this point further, we illuminate the same metalens with several monochromatic source within 200 and 400 nm frequency. The presented metalens exhibit a negative dispersion, higher frequencies have higher focal lengths (Figure S2)^43^. However, Fig. 5c displays a very homogeneous, tight focal spot under broadband illumination. In addition, under 200 nm monochromatic illumination we obtain the focal length of 7.73 $\upmu$m, which is very similar to the focal length under broadband illumination (7.76 $\upmu$m). The results imply that the metalens could be under a non-linear optical effect and the focal length under broadband illumination is determined by lower illumination wavelengths for the presented metalenses. NA of the broadband and monochromatic illumination is calculated as 0.54, and 0.73, respectively. As the NA is directly related with the focal distance, it is expected that the calculated NA would be different for both conditions.

**Figure 6:**
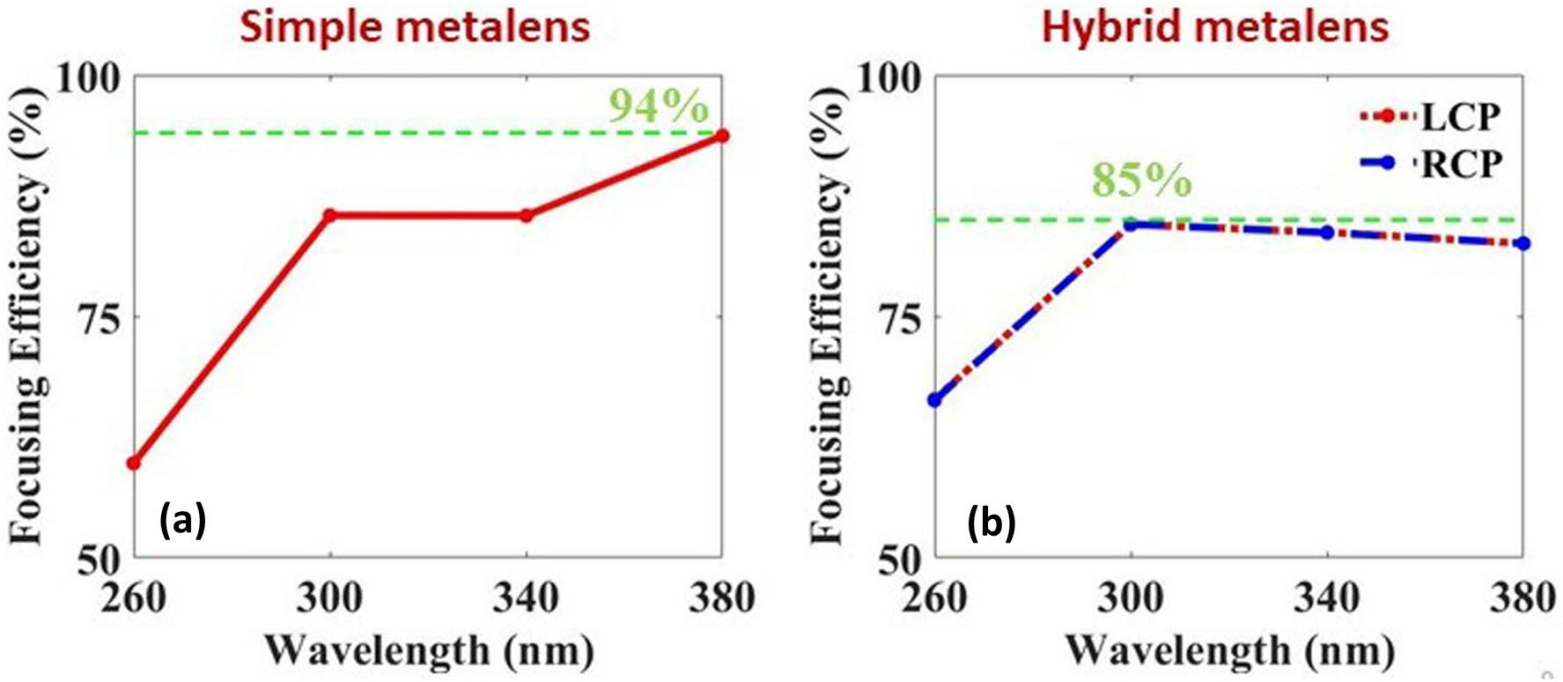
(**a**) Calculated focusing efficiency of the simple metalens with 20 $\upmu$m diameter for the designated wavelengths under RCP illumination, where the horizontal green dashed line shows the maximum value of the FE. (**b**) Focusing efficiency of the hybrid metalens ($R=10\,\upmu$m) under both LCP/RCP illumination, with a maximum value of 85% shown by the dashed line.

The focusing efficiency (FE) of the metalenses are an important parameter that explains the performance of the metalens. It is calculated by taking the ratio of the optical power in the focal region and the incident power. Figure 6 shows the calculated FE for both simple and hybrid metalenses in the UV frequency range (200–400 nm). For the simple metalens the FE are calculated as 60%, 86%, 85%, 94% respectively at the designated wavelengths under RCP illumination as shown in Fig. 6a. The presented efficiencies are the highest reported in the UV range^11,24,25,41,42^. Similarly, for the hybrid case Fig. 6b shows the FE when the hybrid metalens is illuminated by both RCP and LCP light. It is observed that with both RCP/LCP lights, the focusing efficiencies are 66%, 85%, 84%, 83% respectively at the designated wavelengths. The results show that the simple metalens performs slightly stronger FE compared to the hybrid metalens in UV. In comparison to all other reported metalenses in this spectral range, here we report the highest focusing efficiencies for both simple and hybrid metalenses.

## Conclusion

We introduce a low absorption loss and low refractive index dielectric material, MgO, as an ideal candidate for metalenses operating at UV frequencies of 200–400 nm. The reported unit cell design allows the metalens to operate over a broad UV range. In addition, the unit cell can be hybridized to enable lensing with both left and right circularly polarized incident light. Geometric P–B phase method is implemented to achieve the required phase for designing the planar metalens. The polarization conversion efficiency is calculated as high as 94% accompanied by a full 2$\pi$ phase coverage at the designated wavelengths in the UV range. Focusing efficiency, which is the major parameter on the quality of a metalens, is calculated as high as 94% being the best value reported within the UV spectral range so far. The designed metalenses bring high numerical aperture (NA$\approx$0.8) and tight focusing on the focal point. We strongly believe that these highly efficient and broadband metalenses can find usage in numerous applications in the UV range i.e. UV laser, lithography, sterilization, and could pave the way towards the world of miniaturization.

## Supplementary Information

Supplementary Information 1

## References

[1] Yu N, Capasso F (2014). Flat optics with designer metasurfaces. Nat. Mater..

[2] Arbabi A, Horie Y, Bagheri M, Faraon A (2015). Dielectric metasurfaces for complete control of phase and polarization with subwavelength spatial resolution and high transmission. Nat. Nanotechnol..

[3] Kildishev AV, Boltasseva A, Shalaev VM (2013). Planar photonics with metasurfaces. Science.

[4] Jahani S, Jacob Z (2016). All-dielectric metamaterials. Nat. Nanotechnol..

[5] Glybovski SB, Tretyakov SA, Belov PA, Kivshar YS, Simovski CR (2016). Metasurfaces: From microwaves to visible. Phys. Rep..

[6] Khorasaninejad M (2016). Metalenses at visible wavelengths: Diffraction-limited focusing and subwavelength resolution imaging. Science.

[7] Khorasaninejad M (2016). Polarization-insensitive metalenses at visible wavelengths. Nano Lett..

[8] Chen WT, Zhu AY, Sisler J, Bharwani Z, Capasso F (2019). A broadband achromatic polarization-insensitive metalens consisting of anisotropic nanostructures. Nat. Commun..

[9] Wang A, Chen Z, Dan Y (2019). Planar metalenses in the mid-infrared. AIP Adv..

[10] Liang Y (2018). High-efficiency, near-diffraction limited, dielectric metasurface lenses based on crystalline titanium dioxide at visible wavelengths. Nanomaterials.

[11] Guo L (2019). Design of aluminum nitride metalens for broadband ultraviolet incidence routing. Nanophotonics.

[12] Yang H (2017). Polarization-independent metalens constructed of antennas without rotational invariance. Opt. Lett..

[13] Lee G-Y (2018). Complete amplitude and phase control of light using broadband holographic metasurfaces. Nanoscale.

[14] Zheng G (2015). Metasurface holograms reaching 80% efficiency. Nat. Nanotechnol..

[15] Chen WT (2014). High-efficiency broadband meta-hologram with polarization-controlled dual images. Nano Lett..

[16] Wang L (2016). Grayscale transparent metasurface holograms. Optica.

[17] Khorasaninejad M, Ambrosio A, Kanhaiya P, Capasso F (2016). Broadband and chiral binary dielectric meta-holograms. Sci. Adv..

[18] Yoon G (2019). Wavelength-decoupled geometric metasurfaces by arbitrary dispersion control. Commun. Phys..

[19] Zhao Z (2015). Multispectral optical metasurfaces enabled by achromatic phase transition. Sci. Rep..

[20] Wu PC (2017). Versatile polarization generation with aluminium plasmonic metasurface. Nano Lett..

[21] Liang H (2019). High performance metalenses: numerical aperture, aberrations, chromaticity, and trade-offs. Optica.

[22] Chen X (2012). Dual-polarity plasmonic metalens for visible light. Nat. Commun..

[23] Pors A, Nielsen MG, Eriksen RL, Bozhevolnyi SI (2013). Broadband focusing flat mirrors based on plasmonic gradient metasurfaces. Nano Lett..

[24] Hu M, Wei Y, Cai H, Cai Y (2019). Polarization-insensitive and achromatic metalens at ultraviolet wavelengths. J. Nanophoton..

[25] Kanwal S (2020). Polarization insensitive, broadband, near-diffraction-limited metalens in ultraviolet region. Nanomaterials.

[26] Chen WT (2017). Immersion meta-lenses at visible wavelengths for nanoscale imaging. Nano Lett..

[27] Chen WT (2018). A broadband achromatic metalens for focusing and imaging in the visible. Nat. Nanotechnol..

[28] Decker M (2019). Imaging performance of polarization-insensitive metalenses. ACS Photon..

[29] Bayati E, Zhan A, Colburn S, Zhelyeznyakov MV, Majumdar A (2019). Role of refractive index in metalens performance. Appl. Opt..

[30] Yang J, Fan JA (2017). Analysis of material selection on dielectric metasurface performance. Opt. Express.

[31] Ravindra NM, Ganapathy P, Choi J (2007). Energy gap-refractive index relations in semiconductors—An overview. Infrared Phys. Technol..

[32] Taurian OE, Springborg M, Christensen NE (1985). Self-consistent electronic structures of MGO and SRO. Solid State Commun..

[33] Stephens RE, Malitson IH (1952). Index of refraction of magnesium oxide. J. Res. Natl. Bureau Stand..

[34] Aieta F (2012). Aberration-free ultrathin flat lenses and axicons at telecom wavelengths based on plasmonic metasurfaces. Nano Lett..

[35] Khorasaninejad M, Crozier KB (2014). Silicon nanofin grating as a miniature chirality-distinguishing beam-splitter. Nat. Commun..

[36] Palik ED (1985). Handbook of Optical Constants of Solids.

[37] https://refractiveindex.info/?shelf=main&book=MgO&page=Stephens (last accessed on 1/11/2020).

[38] Dong H (2017). Visible-wavelength metalenses for diffraction limited focusing double polarization and vortex beams. Opt. Mater. Express.

[39] Presutti F, Monticone F (2020). Focusing on bandwidth: Achromatic metalens limits. Optica.

[40] Bayati E (2020). Inverse designed metalenses with extended depth of focus. ACS Photon..

[41] Guo L (2018). Design of aluminum nitride metalens in the ultraviolet spectrum. J. Nanophoton..

[42] Kanwal S (2020). High-efficiency, broadband, near diffraction-limited, dielectric metalens in ultraviolet spectrum. Nanomaterials.

[43] Shrestha S, Overvig AC, Lu M, Stein A, Yu N (2018). Broadband achromatic dielectric metalenses. Light Sci. Appl..

