# Supplementary material for: A hybrid broadband metalens operating at ultraviolet frequencies
Source: Sci Rep. 2021 Jan 27;11:2303. doi: 10.1038/s41598-021-81956-4 (PMC7840775; doi:10.1038/s41598-021-81956-4)
Supplement: Supplementary file 1 — Supplementary Information 1 [file 41598_2021_81956_MOESM1_ESM.pdf]

SUPLEMENTARY INFORMATION

A hybrid broadband metalens operating at ultraviolet frequencies

Farhan Ali and Serap Aksu

The optical constants:

The optical constants of glass and MgO are taken from Palik, Stephens et al. respectively [33, 36]. We calculate the refractive index of MgO down to 200 nm using a dispersion relation[37]. Thus, the refractive index used for MgO is not fixed and wavelength dependent. After calculating the refractive index within 200-400nm, we import the data to Lumerical. Below we show the dependance of MgO refractive index on wavelength, which is used for simulations.

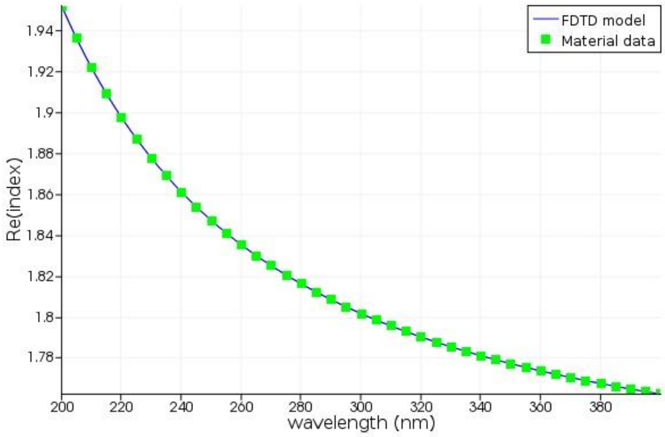

Figure S1. Refractive index- wavelength relation for MgO within related frequency regime. This data is used in FDTD.

Further investigation on metalens under broadband source vs monochromatic source:

In order to understand the focusing properties under broadband light illumination, we simulated the metalenses designed at 300nm (λ_d = 300nm, mentioned on Page 7), and illuminate with different monochromatic light sources within 200-400nm frequency range. As stated in Table S1, the focal length is nearly linearly dependant on incident wavelength. As wavelength moves towards 200nm, the simulated focal length increases, displaying a negative dispersion (Figure S2. The negative dispersion is also observed in references 25 and 42). At 200nm, the simulated focal length is found as ~7.73μm, which is close to the focal length obtained with 200-400 nm broadband illumination (~7.76μm). If the broadband illumination had the same effect as the sum of each monochromatic light, we would not observe an intensity distribution as in Figure 5a. This result implies that a non-linear optical effect could be going on, which worth a further, separate study to explain.

$\lambda_{incident}$ (nm)	Simulated focal length f (μm)
200	7.73
220	6.92
250	5.88
280	5.09
300	4.63
320	4.22
350	3.71
380	3.30
400	3.06

Table S1. Variation of focal length for the metalens designed for 300nm, when illuminated with various monochromatic light within the range of 200-400nm.

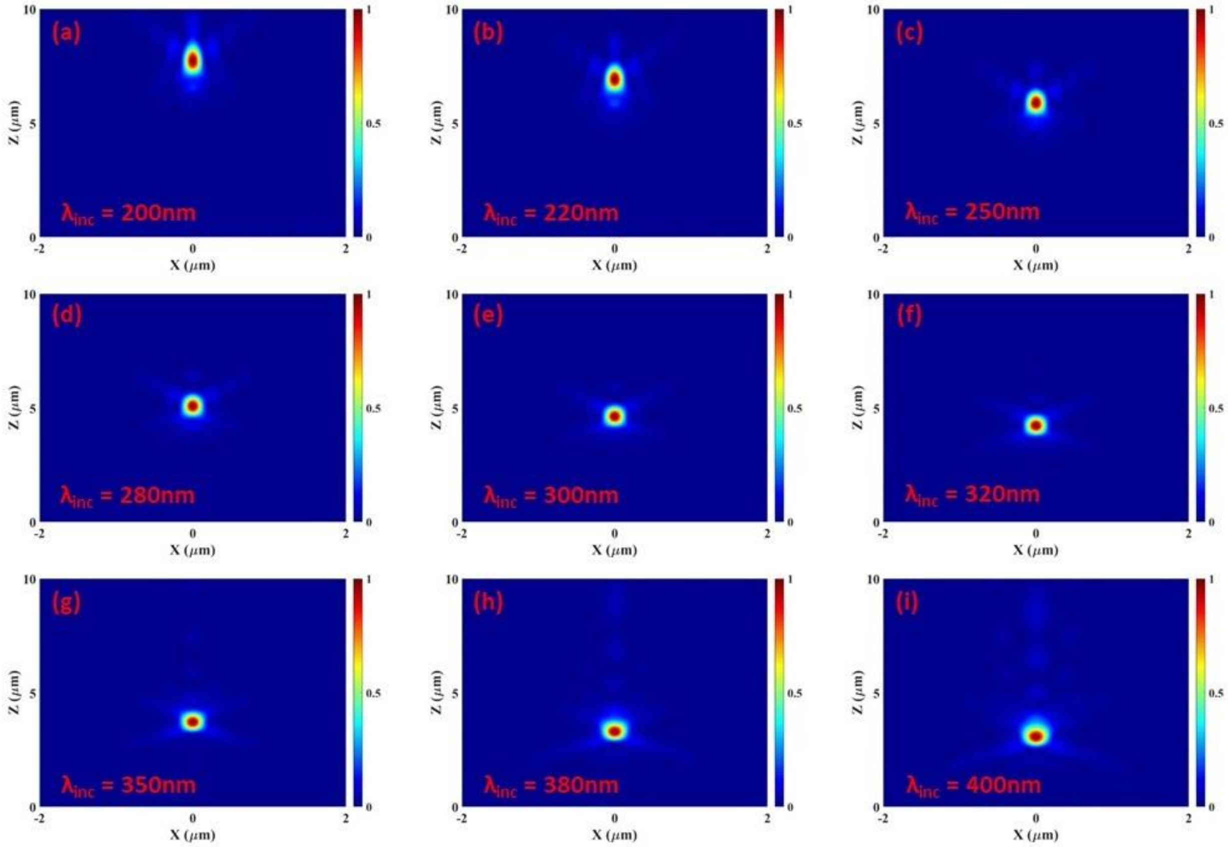

Figure S2. Simulated focal spots (x-z profiles at y=0) for the metalens designed for 300nm, when illuminated with various monochromatic light within the range of 200-400nm.

To further investigate the effect of illumination wavelength, we tested the same metalens design (λ_d = 300nm) under different broadband illumination ranges as shown in Table 2 and Figure S3. When the 275-325 nm broadband light is illuminated on the same metalens (instead of 200-400 nm), we obtain a focal length of 5.19 μm, closer to the focal length obtained under monochromatic 300 nm illumination (4.63 μm). As we further increase the range to 250-350 nm, we see an increase on focal length (5.88 μm). When we change the broadband illumination towards higher wavelengths (250-400nm) we observe a similar focal length (5.88 μm) with

250-350 nm illumination. However, when we change the broadband illumination towards lower wavelengths (225-375nm and 200-250 nm) we observe a significant increase on focal length. This result implies that the focal length for the proposed metalens under broadband illumination (200-400nm) is similar to the focal lengths obtained with monochromatic lower wavelengths (200nm). Hence the focal length under broadband illumination is decided by lower wavelengths.

$\lambda_{incident}$ (nm)	Simulated focal length f (µm)
275-325	5.19
250-350	5.88
250-400	5.88
225-375	6.70
200-250	7.73

Table S2. Variation of focal length for the metalens designed for 300nm, when illuminated with various broadband light within the range of 200-400nm.

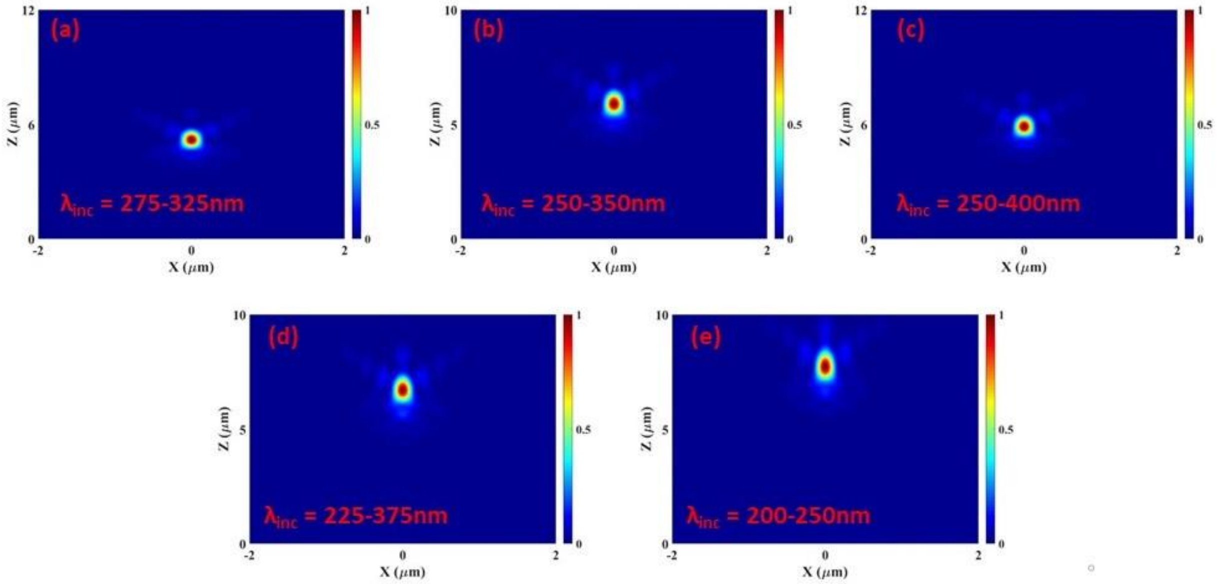

Figure S3. Simulated focal spots (x-z profiles at y=0) for the metalens designed for 300nm, when illuminated with various broadband light within the range of 200-400nm.

Moreover, in order to confirm the focal length increase under broadband illumination compared to monochromatic illumination, we additionally investigate the metalens designed for 380 nm (λ_d = 380nm) illuminated by 350-410 nm broadband light, centering 380 nm wavelength. The focal length under 380 nm monochromatic light is found as 4.56 µm, where under 350-410 nm broadband illumination the focal length is found as 5.01 µm (Table S3). Therefore, similar to the previous case, the broadband illumination results in a higher focal length compared to monochromatic illumination due to the presence of lower (<380 nm) illumination wavelengths.

λ_d (nm)	$\lambda_{incident}$ (nm)	Simulated focal length f (µm)
380	350-410	5.01

Table S3. Simulated focal length for the metalens designed for 380nm, when illuminated with broadband light source 350-410nm, centring 380 nm wavelength.
